# Quantitatively Extreme Sagittal Orientation of the L5-S1 Facet Joints in Adolescent Dysplastic Spondylolisthesis: A Case Report and Mechanistic Considerations

**DOI:** 10.3390/jcm15187311

**Published:** 2026-09-20

**Authors:** Yong-Xi Fan, Jia-Yu Fu, Xiao-Ling Luo, Chang-Sheng Liao, Zhong-Jie Zhou

**Affiliations:** Department of Orthopaedics, West China Hospital, Sichuan University, Chengdu 610041, China; fanyongxi1988@163.com (Y.-X.F.); 15583084027@163.com (J.-Y.F.); 18683614757@163.com (X.-L.L.)

**Keywords:** dysplastic spondylolisthesis, L5-S1 facet joint, facet orientation, adolescent, spinal fusion, case report

## Abstract

**Background/Objectives:** Dysplastic spondylolisthesis is associated with developmental abnormalities of the lumbosacral junction, but the contribution of extremely sagittal L5-S1 facet orientation in an adolescent with an intact pars interarticularis remains incompletely characterized. We aimed to describe this unusual quantitative morphologic phenotype and discuss its possible biomechanical implications. **Methods:** Clinical records and serial imaging of a 14-year-old girl with symptomatic low-grade L5-S1 dysplastic spondylolisthesis were reviewed. Standing radiographs, flexion-extension radiographs, computed tomography (CT), magnetic resonance imaging (MRI), quantitative spinopelvic parameters, intraoperative findings, treatment, and 12-month follow-up were assessed. **Results:** The patient had approximately one year of progressively worsening low back pain and occasional transient right-leg numbness despite more than 8 months of lumbosacral bracing and more than 6 months of supervised physical therapy. Standing radiographs showed Meyerding grade I L5-S1 anterolisthesis and mild thoracolumbar scoliosis. Quantitative spinopelvic assessment showed a pelvic incidence (PI) of 76.63°, pelvic tilt of 22.21°, sacral slope of 54.42°, lumbar lordosis of 59.5°, sagittal vertical axis of 2.67 cm, and a Dubousset lumbosacral angle of 109°. CT demonstrated an intact pars interarticularis, bilateral facet subluxation, and L5-S1 facet angles of 6° on the left and 3° on the right relative to the sagittal plane. Flexion-extension radiographs showed measurable L5-S1 angular motion from −6.7° to +3.5°, while MRI showed no major central canal stenosis, focal disc herniation, or definite neural compression. Posterior reduction and L5-S1 fusion were performed after individualized counseling, and facet subluxation was confirmed intraoperatively. At 6 and 12 months, the patient remained free of recurrent pain or numbness and radiographs showed maintained lumbosacral alignment. **Conclusions:** Quantitatively extreme L5-S1 facet sagittalization accompanied by bilateral facet subluxation, an intact pars, and high-PI low-grade spondylolisthesis represents a distinctive morphologic phenotype. This configuration is biomechanically compatible with reduced resistance to anterior shear, although a single case cannot establish independent causality or justify a general indication for early surgery. Comparative quantitative imaging and longitudinal studies are required.

## 1. Introduction

Developmental or dysplastic spondylolisthesis in children and adolescents encompasses anterior translation at the lumbosacral junction in association with congenital or developmental osseous abnormalities. The Wiltse–Newman system classifies dysplastic spondylolisthesis as type I, whereas more recent frameworks additionally emphasize slip severity, dysplasia, and spinopelvic balance [1,2,3,4]. Typical dysplastic features may include malformation of the L5-S1 facet joints, a trapezoidal L5 vertebral body, rounding or doming of the sacral endplate, and spina bifida occulta. In contrast to isthmic spondylolisthesis, the pars interarticularis may remain intact [1,2].

The L5-S1 facet joints contribute to posterior column stability and resist anterior shear. A more sagittal facet orientation may provide less osseous restraint against forward translation. Associations between sagittal facet morphology and vertebral translation have been studied mainly in adults with degenerative spondylolisthesis, while facet orientation and tropism have also been examined in younger patients with pars lesions [5,6,7,8,9,10]. However, detailed reports correlating bilateral sagittal L5-S1 facet orientation, an intact pars, facet subluxation, and adolescent low-grade spondylolisthesis are uncommon.

We describe an adolescent with Meyerding grade I L5-S1 spondylolisthesis in whom computed tomography (CT) and intraoperative findings demonstrated bilateral facet subluxation and an intact pars interarticularis. The distinctive contribution of this report is the quantitative demonstration of extremely sagittal L5-S1 facet orientation (6° on the left and 3° on the right relative to the sagittal plane), together with high-pelvic-incidence spinopelvic morphology, flexion–extension imaging, and magnetic resonance imaging (MRI) assessment. The aim is to document this morphologic phenotype and discuss its biomechanical and clinical implications without inferring causality from a single case. This report was prepared in accordance with the CARE guideline [11], and the completed CARE checklist is provided as Supplementary File S1.

## 2. Case Presentation

### 2.1. Patient Information and Clinical Findings

A 14-year-old girl presented with intermittent low back pain that had persisted for approximately one year and had gradually worsened. The pain had no consistent relationship to lumbar movement. She also reported occasional transient numbness in the right lower limb; each episode resolved spontaneously and did not materially limit walking or activities of daily living. Before operative decision-making, she underwent a prolonged structured nonoperative program. She wore a lumbosacral brace for more than 8 months and received supervised physical therapy in the Department of Rehabilitation Medicine twice weekly for more than 6 months. Orthopedic outpatient reassessment was performed approximately every 3 months during this period. Despite this prolonged course of conservative management, her low back pain did not achieve durable improvement and progressively worsened, prompting further evaluation and discussion of operative stabilization. The overall clinical course is summarized in Table 1.

Physical examination demonstrated mild lumbosacral tenderness and a palpable step-off. Motor strength, sensation, and deep-tendon reflexes were normal, and the straight-leg-raise test was negative. No persistent neurological deficit was identified.

### 2.2. Diagnostic Assessment

Standing anteroposterior and lateral radiographs showed a mild thoracolumbar scoliotic curve (approximately 20° on re-review) and Meyerding grade I anterolisthesis of L5 on S1. Quantitative standing sagittal parameters were a pelvic incidence (PI) of 76.63°, pelvic tilt (PT) of 22.21°, sacral slope (SS) of 54.42°, lumbar lordosis (LL) of 59.5°, sagittal vertical axis (SVA) of 2.67 cm, and Dubousset lumbosacral angle (Dub-LSA) of 109°. Because the slip was low-grade and PI exceeded 60°, the morphology corresponds to Spinal Deformity Study Group (SDSG) type 3 (high-PI low-grade spondylolisthesis) [3,4]. Flexion–extension radiographs were also available. The supplied measurements recorded L5-S1 segmental angles of −6.7° and +3.5°, respectively, corresponding to a 10.2° angular excursion. We report this as measurable segmental mobility rather than as proof of pathologic dynamic instability because a validated instability threshold for this specific dysplastic adolescent phenotype is not established. The quantitative preoperative radiographic parameters are summarized in Table 2.

**Figure 1:**
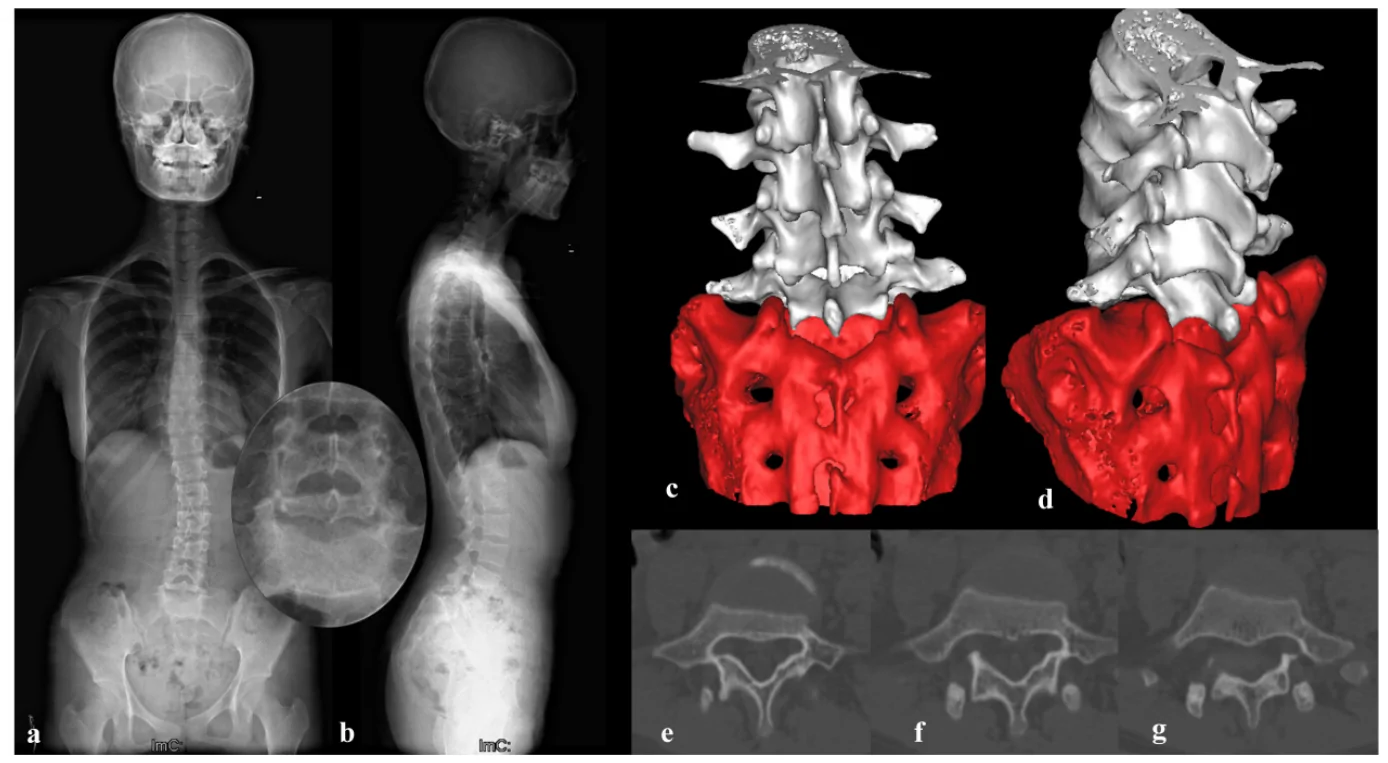
Preoperative radiographic and computed tomography (CT) findings. (**a**) Standing anteroposterior full-spine radiograph showing a mild thoracolumbar scoliotic curve. (**b**) Standing lateral radiograph showing Meyerding grade I anterolisthesis of L5 on S1 and mild rounding of the sacral endplate. (**c**,**d**) Posterior and oblique three-dimensional CT reconstructions showing preserved pars interarticularis morphology and anterior translation/subluxation of the L5 inferior facets relative to the S1 superior facets. (**e**–**g**) Consecutive axial CT images demonstrating nearly sagittal orientation of the L5-S1 facet joints.

**Figure 2:**
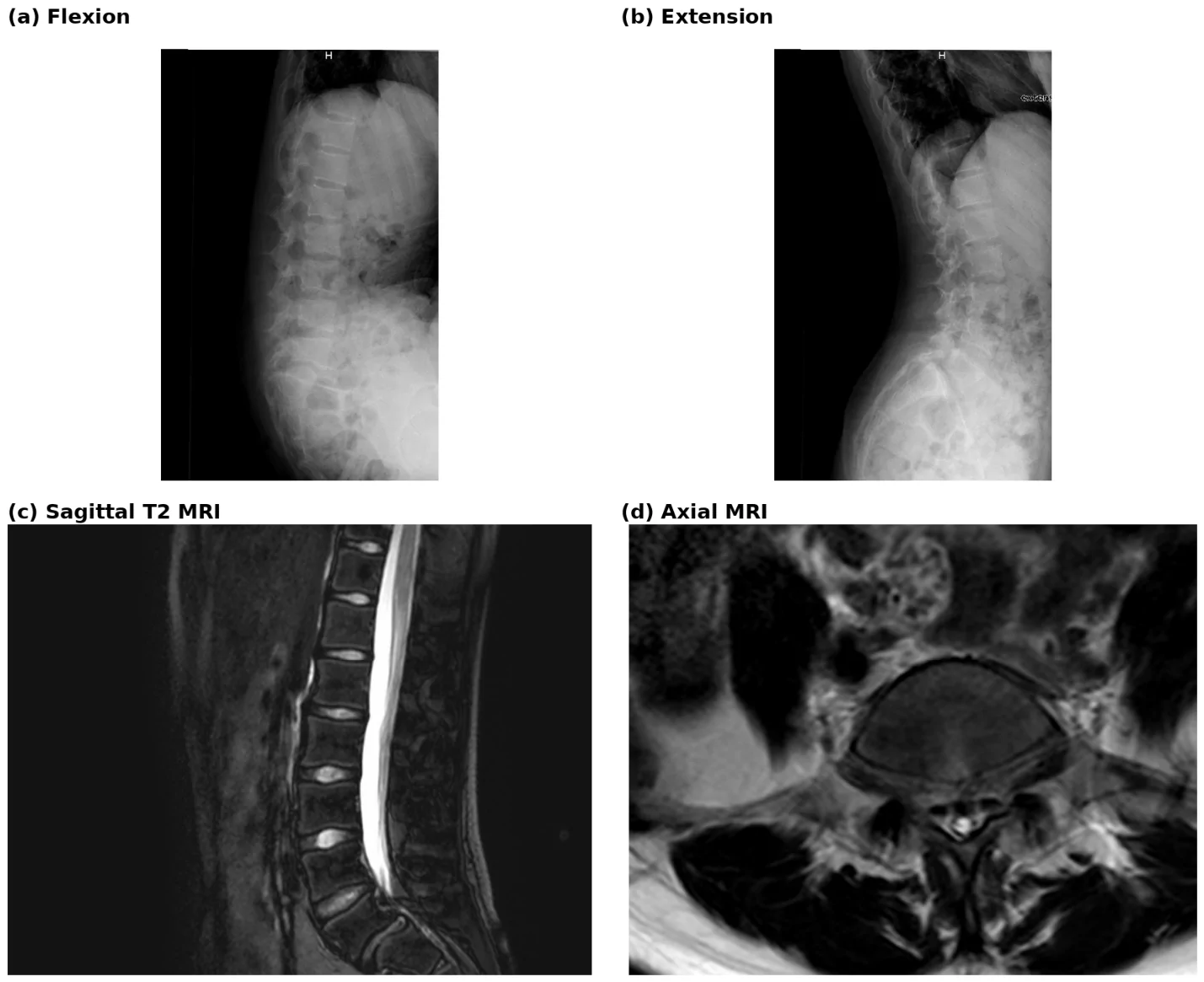
Flexion–extension radiographs and preoperative magnetic resonance imaging (MRI). (**a**,**b**) Flexion and extension lateral radiographs demonstrate measurable motion at L5-S1; the supplied segmental-angle measurements changed from −6.7° to +3.5° (10.2° excursion). This finding is reported as segmental mobility and is not interpreted as a validated threshold of pathologic instability. (**c**) Sagittal T2-weighted MRI and (**d**) axial MRI demonstrate the low-grade L5-S1 slip without a large focal disc extrusion, major central canal stenosis, or definite focal neural compression.

CT demonstrated preserved L5 morphology and an intact pars interarticularis. The superior sacral endplate was mildly rounded. Most notably, the bilateral L5-S1 facet joints were oriented nearly parallel to the sagittal plane, and the inferior articular processes of L5 were translated anteriorly relative to the superior articular processes of S1, consistent with facet subluxation (Figure 1). Facet orientation was measured on axial CT as the angle between the facet joint line and the midsagittal plane; the values were 6° on the left and 3° on the right, with smaller values indicating a more sagittal orientation. For context, a young control cohort measured with the same sagittal-plane convention had a mean L5-S1 facet angle of approximately 42.2° ± 4.9° [12]. Magnetic resonance imaging (MRI) demonstrated the low-grade L5-S1 slip without a large disc extrusion, major central canal stenosis, or definite focal neural compression (Figure 2).

The patient was considered to have a developmental/dysplastic L5-S1 slip characterized predominantly by abnormal facet morphology rather than an isthmic defect. The high PI (76.63°), facet subluxation, extremely sagittal facet angles, and reduced Dub-LSA (109°) were interpreted as markers of a mechanically vulnerable lumbosacral junction. Published data using the Dubousset method report mean values of approximately 119.0° in controls and 110.3° in low-grade spondylolisthesis, placing the present measurement within the low-grade spondylolisthetic range [13]. An exact standardized slip percentage was not available from the source dataset, so Meyerding grade I was retained as the slip descriptor.

**Table 1:** Clinical timeline. Abbreviations: CT, computed tomography; MRI, magnetic resonance imaging; PI, pelvic incidence; SDSG, Spinal Deformity Study Group; Dub-LSA, Dubousset lumbosacral angle.

Time Point	Clinical Course	Key Findings or Actions
Approximately 12 months before presentation	Onset of intermittent low back pain	Symptoms persisted and gradually worsened
Before presentation	Occasional transient right-leg numbness	Episodes resolved spontaneously; walking and daily activities were preserved
At presentation	Clinical and neurological examination	Mild lumbosacral tenderness and palpable step-off; normal motor, sensory, and reflex findings; negative straight-leg-raise test
Preoperative conservative treatment	Bracing, supervised rehabilitation, and serial orthopedic follow-up	Lumbosacral brace for >8 months; supervised physical therapy twice weekly in the Department of Rehabilitation Medicine for >6 months; orthopedic reassessment approximately every 3 months; pain remained persistent and progressively worsened
Diagnostic assessment	Standing radiographs, flexion–extension radiographs, CT, and MRI	Meyerding grade I L5-S1 anterolisthesis; PI 76.63° (SDSG type 3); Dub-LSA 109°; intact pars; facet angles 6° left/3° right with bilateral facet subluxation; measurable L5-S1 angular mobility; no major compressive lesion on MRI
Intervention	Posterior reduction and L5-S1 fusion	Facet subluxation confirmed intraoperatively
Immediate postoperative period	Routine recovery	No perioperative complication documented; lumbosacral alignment restored on radiographs
6- and 12-month follow-up	Clinical and radiographic reassessment	No recurrent pain or numbness; maintained alignment

**Table 2:** Quantitative preoperative radiographic parameters obtained on re-review of the available imaging. Abbreviations: PI, pelvic incidence; PT, pelvic tilt; SS, sacral slope; LL, lumbar lordosis; SVA, sagittal vertical axis; SDSG, Spinal Deformity Study Group.

Parameter	Value	Interpretation/Context
Meyerding grade	Grade I	Low-grade slip
Pelvic incidence (PI)	76.63°	High PI; with low-grade slip, SDSG type 3 [3,4]
Pelvic tilt (PT)	22.21°	Standing sagittal parameter
Sacral slope (SS)	54.42°	Standing sagittal parameter
Lumbar lordosis (LL)	59.5°	Standing sagittal parameter
Sagittal vertical axis (SVA)	2.67 cm	Global sagittal alignment parameter
Dubousset lumbosacral angle	109°	Reduced relative to controls; close to published low-grade values [13]
L5-S1 facet angle	Left 6°; right 3°	Angle to sagittal plane; extremely sagittal relative to young controls (~42.2° ± 4.9°) [12]
L5-S1 flexion–extension segmental angle	−6.7° to +3.5°	10.2° excursion; reported as mobility, not a validated instability threshold

### 2.3. Therapeutic Intervention

Nonoperative management is generally considered first-line for neurologically intact adolescents with low-grade spondylolisthesis [1,14,15]. In the present case, operative treatment was considered only after a prolonged structured conservative program. The patient had worn a lumbosacral brace for more than 8 months and had attended supervised physical therapy in the Department of Rehabilitation Medicine twice weekly for more than 6 months, with orthopedic follow-up approximately every 3 months. Nevertheless, her low back pain persisted and progressively worsened. The decision for surgery was therefore not based on the Meyerding grade I slip alone. Rather, it reflected inadequate symptomatic response to prolonged conservative care together with a cluster of concordant structural findings: a palpable step-off, bilateral facet subluxation confirmed on CT, quantitatively extreme sagittal facet orientation, high-PI SDSG type 3 morphology, and a reduced Dubousset lumbosacral angle. Flexion–extension imaging demonstrated measurable segmental motion, although this finding was not interpreted as independent proof of pathological dynamic instability; MRI did not show a major compressive lesion that could otherwise explain the symptoms. After discussion of continued nonoperative care versus surgical stabilization with the patient and her legal guardian, posterior pedicle-screw instrumentation, reduction, and L5-S1 interbody fusion were selected. Intraoperative inspection confirmed subluxation of the L5-S1 facet articulation. This individualized decision should not be generalized as an indication for early fusion in all adolescents with low-grade spondylolisthesis.

### 2.4. Follow-Up and Outcomes

**Figure 3:**
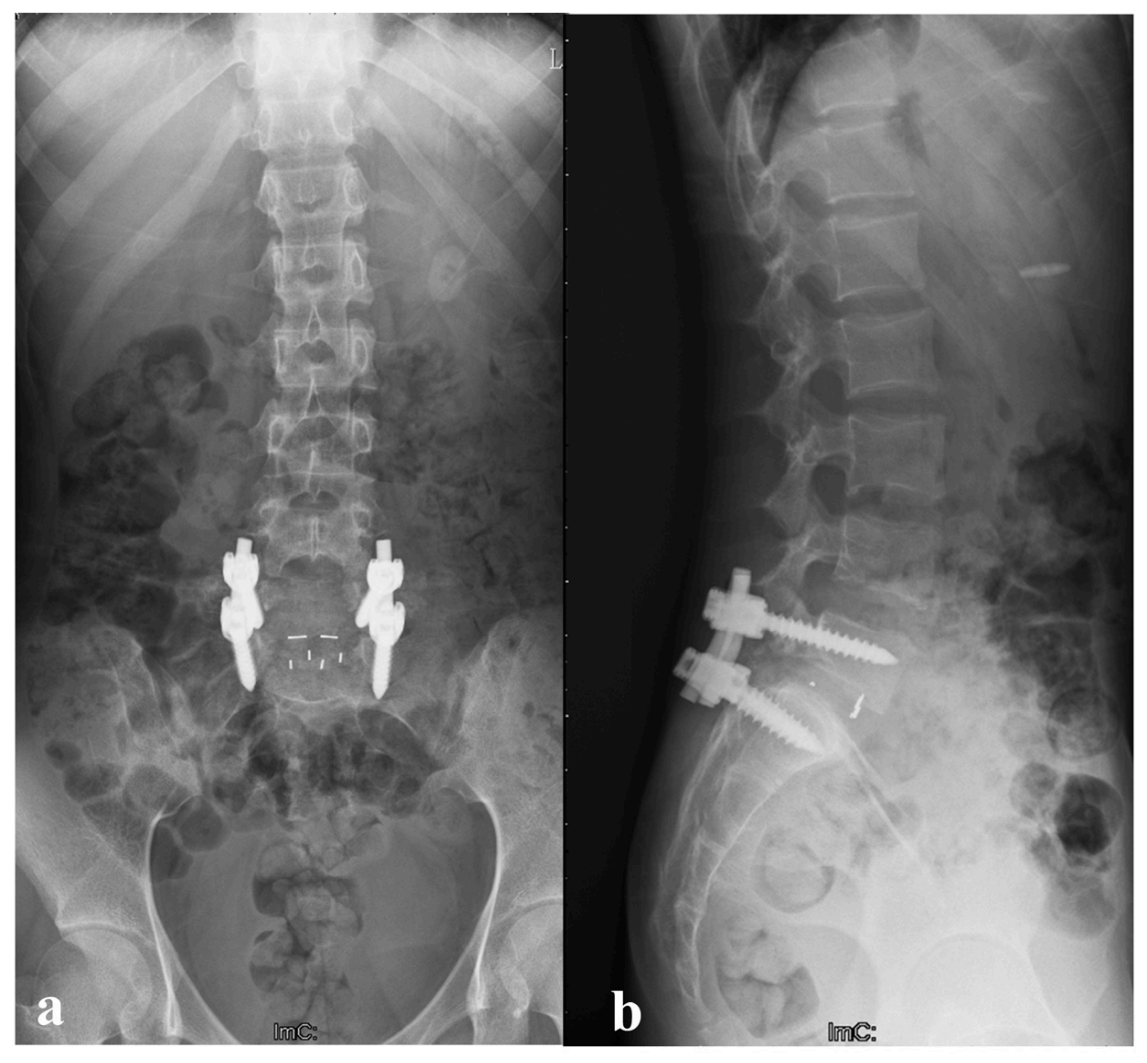
Postoperative anteroposterior (**a**) and lateral (**b**) radiographs showing L5-S1 posterior instrumentation and interbody fusion with restoration of lumbosacral alignment.

The postoperative course was uncomplicated. Early anteroposterior and lateral radiographs showed L5-S1 instrumentation and restoration of lumbosacral alignment (Figure 3). At 6 and 12 months after surgery, the patient reported no recurrence of low back pain or right-leg numbness. Follow-up radiographs demonstrated maintained alignment. Patient-reported outcome scores were not available.

## 3. Discussion

### 3.1. Morphologic and Biomechanical Interpretation

The principal observation in this case is the concordance among quantitative axial CT, three-dimensional CT reconstruction, and intraoperative findings: both L5-S1 facet joints were extremely sagittal (6° left and 3° right relative to the sagittal plane), the pars interarticularis was preserved, and the facets were subluxated in the direction of vertebral translation. This combination provides a plausible mechanical explanation for reduced posterior restraint at the lumbosacral junction. Importantly, the quantitative values are not merely descriptive. In a young control cohort, the mean L5-S1 facet angle measured relative to the sagittal plane was approximately 42.2° ± 4.9° [12], making the 3–6° values in this patient markedly more sagittal than typical reference values.

Facet orientation affects the direction in which the posterior elements resist load. At L5-S1, a more coronal orientation can oppose anterior shear, whereas sagittalization permits relatively greater anteroposterior translation. Studies of adult degenerative spondylolisthesis have repeatedly reported associations between sagittal facet orientation and vertebral slip [5,6,7,8], and younger cohorts have demonstrated relationships between facet morphology, tropism, and pars-related disorders [9,10,12]. These data support biomechanical plausibility but do not resolve whether sagittal orientation is a primary developmental abnormality, an adaptive change, or one component of a multifactorial process.

In the present patient, the young age, intact pars, bilateral morphology, and intraoperative facet subluxation favor a developmental interpretation rather than an adult degenerative mechanism. The quantitative spinopelvic profile provides additional context: PI was 76.63°, PT was 22.21°, SS was 54.42°, LL was 59.5°, and SVA was 2.67 cm. With a low-grade slip and PI >60°, the patient fits SDSG type 3, the high-PI or shear-type low-grade pattern [3,4]. The Dub-LSA was 109°, which is lower than the mean value reported in controls (119.0° ± 8.3°) and close to the published low-grade spondylolisthesis mean (110.3° ± 13.3°) [13]. Mild rounding of the sacral endplate may represent an associated dysplastic feature or remodeling related to chronic translation [16]. These findings describe the mechanical environment but do not establish a causal sequence.

### 3.2. Classification and Clinical Implications

The original Wiltse–Newman classification already recognizes dysplastic spondylolisthesis as a category that may include congenital abnormalities of the lumbosacral facets [2]. Therefore, the current finding is better regarded as a potentially under-recognized morphologic phenotype within the dysplastic spectrum than as proof of a wholly new etiologic subclass. The Spinal Deformity Study Group framework incorporates slip grade, pelvic incidence, sacropelvic balance, and global alignment [3,4]. Because this patient had a low-grade slip and PI of 76.63°, she is classified as SDSG type 3. The balanced/retroverted pelvic subcategories are principally used for high-grade slips and were therefore not applied here. The Lamartina L5 severity index was also not emphasized because the present slip was Meyerding grade I. Future classification research could evaluate whether quantitative facet angle adds reproducible diagnostic or prognostic information beyond established spinopelvic variables.

This case should not be interpreted as supporting routine early surgery for all adolescents with low-grade spondylolisthesis. Most low-grade slips are initially managed nonoperatively, and operative treatment is generally individualized according to symptoms, progression, neurological findings, deformity, and response to conservative care [1,14,15,17]. In the present patient, a prolonged nonoperative program had already been completed: bracing for more than 8 months, supervised physical therapy twice weekly in the Department of Rehabilitation Medicine for more than 6 months, and orthopedic reassessment approximately every 3 months. Persistent and progressively worsening pain despite this treatment was considered together with bilateral facet subluxation, extremely sagittal facet orientation, high-PI shear-type morphology, and the reduced Dubousset lumbosacral angle. Flexion–extension radiographs showed measurable angular motion but did not independently establish pathological dynamic instability, and MRI excluded a major compressive lesion as the primary explanation for the intermittent numbness. Thus, stabilization was selected only after prolonged conservative treatment had failed to provide durable symptomatic improvement and after shared decision-making with the patient and her legal guardian.

### 3.3. Strengths and Limitations

A strength of this report is the direct correlation of standing radiographs, quantitative spinopelvic measurements, flexion–extension imaging, MRI, axial and three-dimensional CT, intraoperative anatomy, and 12-month follow-up. The imaging clearly demonstrates preservation of the pars, quantitatively extreme facet sagittalization, and facet subluxation, helping distinguish the observed pattern from a typical isthmic lesion.

Several limitations are important. First, a single case cannot establish prevalence, temporal sequence, independent causality, or a new classification category. Second, although facet angles and major spinopelvic parameters could be quantified on re-review, an exact standardized slip percentage and longitudinal DICOM-based measurements were not available. Third, flexion–extension radiographs demonstrated a 10.2° L5-S1 angular excursion, but no validated threshold allows this value alone to be labeled pathologic instability in this specific dysplastic adolescent phenotype. Fourth, validated patient-reported outcome measures were not documented. Finally, 12 months is sufficient to describe early clinical recovery but not long-term fusion status, adjacent-segment effects, or slip recurrence. Comparative imaging studies and longitudinal cohorts are needed to test the proposed biomechanical relationship.

## 4. Conclusions

Quantitatively extreme sagittal orientation of the L5-S1 facet joints (6° left and 3° right relative to the sagittal plane), accompanied by bilateral facet subluxation and an intact pars interarticularis, was observed in an adolescent with low-grade dysplastic spondylolisthesis. The patient also had a high-PI SDSG type 3 spinopelvic profile and a Dubousset lumbosacral angle of 109°. This morphology may reduce posterior resistance to anterior shear and contribute to lumbosacral mechanical vulnerability. The case supports deliberate assessment of facet orientation and spinopelvic parameters when an adolescent slip is not explained by a pars defect, but it does not establish an independent etiology, a new classification subtype, or a general indication for early surgery. Larger quantitative and longitudinal studies are required.

## Data Availability

The clinical information supporting this case report is contained within the article. Additional de-identified information may be available from the corresponding author upon reasonable request, subject to institutional approval and patient privacy requirements.

## Supplementary Materials

The following supporting information can be downloaded at: https://www.mdpi.com/article/10.3390/jcm15187311/s1, Supplementary File S1: CARE checklist.
