# Supplementary material for: Quantitatively Extreme Sagittal Orientation of the L5-S1 Facet Joints in Adolescent Dysplastic Spondylolisthesis: A Case Report and Mechanistic Considerations
Source: J Clin Med. 2026 Sep 20;15(18):7311. doi: 10.3390/jcm15187311 (PMC13607587; doi:10.3390/jcm15187311)
Supplement: Supplementary file 1 [file jcm-15-07311-s001.zip › jcm-4512971-supplementary.pdf]

CARE Checklist of Information to Include When Writing a Case Report

Manuscript: Quantitatively Extreme Sagittal Orientation of the L5-S1 Facet Joints in Adolescent Dysplastic Spondylolisthesis: A Case Report and Mechanistic Considerations

Topic	Item	Checklist Item Description	Reported on Page/Section	Status
Title	1	The diagnosis or intervention of primary focus followed by the words "case report".	Page 1	Yes
Key Words	2	2 to 5 key words that identify diagnoses or interventions in this case report, including "case report".	Page 2	Yes
Abstract	3a	Introduction: What is unique about this case and what does it add to the scientific literature?	Pages 1–2	Yes
Abstract	3b	Main symptoms and/or important clinical findings.	Pages 1–2	Yes
Abstract	3c	The main diagnoses, therapeutic interventions, and outcomes.	Pages 1–2	Yes
Abstract	3d	Conclusion—What is the main "take-away" lesson(s) from this case?	Pages 1–2	Yes
Introduction	4	One or two paragraphs summarizing why this case is unique (may include references).	Pages 2–3	Yes
Patient Information	5a	De-identified patient specific information.	Section 2.1, Page 3	Yes
Patient Information	5b	Primary concerns and symptoms of the patient.	Section 2.1, Page 3	Yes
Patient Information	5c	Medical, family, and psycho-social history including relevant genetic information.	Not specifically reported in the available source record	Not reported
Patient Information	5d	Relevant past interventions with outcomes.	Section 2.1, Page 3	Yes
Clinical Findings	6	Describe significant physical examination and important clinical findings.	Section 2.1, Page 3	Yes
Timeline	7	Historical and current information from this episode of care organized as a timeline.	Table 1, Pages 4–5	Yes
Diagnostic Assessment	8a	Diagnostic testing (such as physical examination, laboratory testing, imaging, surveys).	Section 2.2 and Figures 1–2, Pages 3–7	Yes
Diagnostic Assessment	8b	Diagnostic challenges (such as access to testing, financial, or cultural).	No specific diagnostic-access, financial, or cultural challenge was reported	Not applicable
Diagnostic Assessment	8c	Diagnosis (including other diagnoses considered).	Section 2.2, Pages 3–4	Yes
Diagnostic Assessment	8d	Prognosis (such as staging in oncology) where applicable.	Not applicable to this case	N/A
Therapeutic Intervention	9a	Types of therapeutic intervention (such as pharmacologic, surgical, preventive, self-care).	Section 2.3, Page 8	Yes
Therapeutic Intervention	9b	Administration of therapeutic intervention (such as dosage, strength, duration).	Section 2.3, Page 8	Yes—surgical intervention described
Therapeutic Intervention	9c	Changes in therapeutic intervention (with rationale).	No change in the selected operative strategy was reported	N/A
Follow-up and Outcomes	10a	Clinician and patient-assessed outcomes (if available).	Section 2.4, Page 8	Yes
Follow-up and Outcomes	10b	Important follow-up diagnostic and other test results.	Section 2.4 and Figure 3, Pages 8–9	Yes
Follow-up and Outcomes	10c	Intervention adherence and tolerability (How was this assessed?).	Not applicable to a single definitive surgical intervention; postoperative course was uncomplicated	N/A
Follow-up and Outcomes	10d	Adverse and unanticipated events.	Section 2.4, Page 8	Yes—none documented
Discussion	11a	A scientific discussion of the strengths AND limitations associated with this case report.	Section 3.3, Page 12	Yes
Discussion	11b	Discussion of the relevant medical literature with references.	Sections 3.1–3.2, Pages 9–11	Yes
Discussion	11c	The scientific rationale for any conclusions (including assessment of possible causes).	Sections 3.1–3.2, Pages 9–11	Yes
Discussion	11d	The primary "take-away" lessons of this case report (without references) in a one paragraph conclusion.	Section 4, Page 12	Yes
Patient Perspective	12	The patient should share their perspective in one to two paragraphs on the treatment(s) they received.	A patient-perspective paragraph was not available	Not reported
Informed Consent	13	Did the patient give informed consent? Please provide if requested.	Informed Consent Statement, Page 13	Yes

Note: This checklist reflects the content of the final proof-correction version. Items not available in the source record are reported transparently as not reported or not applicable rather than reconstructed.
